# Supplementary material for: SS-VIME: a single-source virome-microbiome extraction protocol toward comprehensive soil community analysis
Source: Microbiol Spectr. 2026 Mar 24;14(5):e03323-25. doi: 10.1128/spectrum.03323-25 (PMC13141886; doi:10.1128/spectrum.03323-25)
Supplement: File S1 — SS-VIME: Single-Source Virome-Microbiome Detailed Extraction Protocol for simultaneous profiling of viral, bacterial, and fungal communities from a single soil sample. [file spectrum.03323-25-s0001.pdf]

SS-VIME: Single-Source Virome-Microbiome Extraction Protocol
For simultaneous profiling of viral, bacterial, and fungal communities from a single soil sample

DOI: dx.doi.org/10.17504/protocols.io.kxygxp64zl8j/v1

Naser Poursalavati, PhD Candidate in Soil Virology
Saint-Jean-sur-Richelieu Research and Development Centre, Agriculture and Agri-Food Canada
Department of Biology, Université de Sherbrooke

Background

This extended cellulose column chromatography protocol represents an advancement based on our previous methods developed for soil samples: an improved total nucleic acids extraction technique [1] and a method for capturing double-stranded RNA (dsRNA) from soil extracts using cellulose column chromatography [2]. The latter was subsequently adapted for use in other media, such as fungal mycelium and plant tissues [3-5]. This updated protocol now captures not only dsRNA but also other microbiome components from a single sample, enabling simultaneous profiling of both the virome and other microbiome members.
The DNA component has been successfully used to capture bacterial and fungal profiles, with potential to include other members like arbuscular mycorrhizal fungi (AMF). RNA can also be recovered separately using this method, making it suitable for evaluating functional aspects of the soil microbiome. This protocol is highly cost-effective, while providing excellent yield. For the first time, it allows the capture of both DNA and dsRNA in a unified process, offering a comprehensive view of the soil's viral and microbial communities.

Prior to Extraction Day

- **Sample Storage**: Immediately upon arrival in the lab, freeze soil samples at -80°C (or -20°C for storage durations under 6 months). This step is crucial to preserve microbial community integrity.
- **Prepare Bead Tubes**: Autoclave 0.1 mm and 3 mm silica beads separately. Under a laminar flow hood, carefully transfer 2 ml of 0.1 mm beads and 10 of the 3 mm beads into 15 ml tubes. Store the prepared bead tubes at -20°C. Prioritize this preparation step to ensure the beads are ready when needed.
- **Weigh Soil Samples**: Once bead tubes are completely frozen and cooled (at least 2 hours at -20°C), carefully weigh 1.5 - 2 grams of mineral soil, or 1 - 1.5 grams of organic or peat soil. Adjust the weight based on the soil texture and the presence of large particles (e.g., gravel, stones). Avoid excessive drying during weighing, as this can impact the microbial community. To ensure accurate measurement, remove stones or debris from the soil using a sterilized toothpick or spatula.

✓ **Best Practice**: Prepare bead tubes in advance and store them at -20°C. Upon receiving the soil samples in the lab, promptly weigh the samples and transfer them to the pre-frozen bead tubes. Keep the tubes frozen until the day of extraction.

Day of Extraction

- **Prepare Chromatography Washing Buffer**: Prepare a fresh batch of chromatography washing buffer according to the buffer section of the protocol.
- **Prepare Slurry**: Combine 1.6 grams of Sigma cellulose powder with 16 ml of chromatography washing buffer. Shake the mixture for 15 minutes at 300 rpm.
- **Water Bath Setup:** Heat a water bath to a stable temperature of 37°C.
- **Centrifuge Preparation:** Set the centrifuge with a 15 ml bucket to 4°C. Simultaneously, place the 5 ml rotor in the refrigerator to ensure it reaches 4°C.
- **Ice Preparation:** Fill a Styrofoam container with ice for cooling steps and incubation processes.

✓ ***Important Notes:***
This protocol is optimized for processing up to 8 samples in parallel, which is the recommended maximum. The procedure will yield 16 final outputs (DNA and dsRNA) and can be completed in under 8 hours.

Lysing Step

- **For each frozen bead tube containing soil, add the following:**
  - 4 ml of PVP-amended CTAB buffer
  - 100 µl of β-mercaptoethanol
  - 2 ml of water-saturated phenol (pH 6.5) and 2 ml of chloroform – isoamyl alcohol (24:1). Alternatively, use 4 ml of phenol:chloroform:isoamyl alcohol (PCI mixture 25:24:1) if unavailable separately.
- **Seal Tubes:** Seal the tubes with Parafilm and ensure the caps are tightly secured. If the tube caps have built-in seals, this step can be skipped, though it is recommended for safety.
- **MiniG Homogenization:** Homogenize the tubes using a MiniG homogenizer at 1500 rpm for 1.5 minutes, pausing for 1 minute on ice after every 30 seconds of homogenization. For organic and peat soils, start with two rounds of 15 seconds each, with 1 minute of cooling in between. If the yield is insufficient, gradually increase the homogenization time, up to a maximum of 1.5 minutes.
- **High-Speed Vortexing:** After cooling for 1 minute, vortex the tubes at high speed for 1 minute, then for 2 minutes, and finally for 5 minutes, with a 1-minute cooling interval between each vortexing period. For organic and peat soils, reduce the final vortexing step to 2 minutes, ensuring the vortex speed does not exceed 1500 rpm.
- **Rest Period:** Allow the tubes to rest on the vortex for 10 minutes, followed by an additional 1-minute vortexing at high speed.
- **Centrifugation of Lysing Tubes:** After the final 1-minute vortex, centrifuge the lysing tubes at 5000 rpm for 5 minutes.

During The 10-Minute Rest: Centrifuge the slurry at 2500 rpm for 1 minute and discard the supernatant. Add another 16 ml washing buffer and shake for 10 minutes at 300 rpm.

During The Centrifugation Step: The slurry shaking for 10 minutes should be completed. Proceed with the next step while the lysing tubes are centrifuging.

Chromatography Step

- **Column Preparation:** Transfer 2 ml of slurry to 2ml Pierce™ centrifuge columns and perform a pulse spin for 30 seconds, then discard the liquids. This pulse spin should be done immediately after completing the centrifugation of the lysing tubes.
- **Add Aqueous Phase:** Add 3 ml of the lysing tubes' aqueous phase to each column.
- **Ethanol Addition:** Add 580 µl of anhydrous ethanol to each column to achieve a final concentration of approximately 16.2%.
- **Column Shaking:** Seal both ends of the columns and shake thoroughly for 10 minutes at 200 rpm. Periodically rotate the columns during shaking to ensure even distribution of the cellulose.
- **Initial Collection:** loosen the cap and place the column inside a fresh 15 ml tube, then pulse spin for 30 seconds. Collect all liquid into a screw-capped 5 ml tube and store at -20°C for total DNA analysis.
- **Washing Buffer Addition:** Add 2 ml of chromatography washing buffer to the columns, briefly shake or vortex to dissolve the cellulose, and incubate for 1 minute.
- **Wash and Disposal:** Transfer the column to the same 15 ml tube and pulse spin for 30 seconds. Discard the liquid. If total RNA processing is required, save this wash elute for RNA analysis.
- **Column Drying:** Discard the liquid and dry the columns by performing a 30-second pulse spin with the cap open.
- **Final Elution:** Add 2 ml of pure water, gently shake to dissolve the cellulose, and incubate for 2 minutes. Then, place each column into a new 15 ml tube.
- **Optional dsRNA Maximization:** Transfer any pelleted cellulose from the 15 ml tube to the column.
- **dsRNA Collection:** Perform a 30-second pulse spin and transfer the liquid to a screw-capped 5 ml tube for dsRNA analysis.

Digestion and Purification Step

- **Enzyme Treatment Setup:** Add 195-200 µl of 10x DNase buffer to each 5 ml eluted tube, followed by 3 µl of DNase I (1 U/µl) and 5 µl of RNase T1 (10 U/µl). Hand shake the mixture for 15 seconds, then incubate the tubes at 37°C for 30 minutes in a water bath.

✓ ***Important Note***:
The enzyme quantities provided are optimized for the extraction process. However, after completing the protocol, it is recommended to quantify the yield using Qubit, allowing for

adjustments to enzyme levels and tailoring of the extraction process for specific soil types if necessary.

- **PCI Extraction:** Add an equal volume (2 ml) of PCI to each tube. Hand shake for 15 seconds, then centrifuge at 18,000 G for 10 minutes.
- **Thaw DNA Tubes:** While the centrifugation is running, remove DNA tubes from -20°C to thaw completely.
- **Transfer Aqueous Phase:** After centrifugation, transfer 2 aliquots of ~950 µl from each PCI tube into new screw-capped 5 ml tubes.
- **Chloroform Purification:** Add an equal volume of chloroform to all DNA, dsRNA, and RNA tubes (if collected). Hand shake for 15 seconds, then centrifuge at 18,000 G for 15 minutes.
- **Careful Aqueous Transfer:** Carefully transfer a total of two aliquots of 830 µl from the dsRNA portion (up to ~1.7 ml total) into a 5 ml Eppendorf tube, and combine three aliquots of 835 µl from the DNA portion (~2.5 ml total) into another Eppendorf tube using a P1000 pipette.

✓ ***Important Note***:
If the upper phase is insufficient, take as much as you can while avoiding contact with or disturbance of the interphase during the transfer.

Precipitation Step

- **Add PEG-NaCl:** Add an equal volume of PEG-NaCl to each tube, close the cap, and hand-shake for 15 seconds.
- **Incubate on Ice:** Incubate the tubes on ice for at least 25 minutes (recommended 55 minutes to 1 hour). If possible, store in the fridge at 4°C with the ice.
- **Pellet Precipitation:** Centrifuge the tubes at 18,000 G for 15 minutes. Carefully pour off the supernatant, then use a P1000 pipette to remove any remaining liquid while keeping the tube inverted. Avoid touching the pellet.

Elution and Purification

- **Ethanol Wash:** Add 2 ml of 75% cold ethanol to each tube, close the cap, and hand shake thoroughly to detach the pellet as much as possible.
- **Ethanol Spin:** Centrifuge at 18,000 G for 10 minutes, then immediately pour off the ethanol and air-dry the pellet for 10 minutes.
- **Prepare PCR Tubes:** While drying, prepare PCR tubes for elution collection.
- **Elute DNA and dsRNA:** After drying, elute the DNA pellet using 50 µl of TE buffer. For dsRNA, use 30 µl of TE buffer and transfer the eluted samples to new PCR tubes.
- **Clarification Spin:** If the solution appears cloudy or contains cellulose traces, perform a high-speed spin and transfer the clarified solution to new PCR tubes.

Qualification and Quantification

- **Nanodrop Analysis:** Perform a Nanodrop analysis using 2 µl for each DNA and dsRNA component. Optionally, use Qubit for more precise quantification.
- **Agarose Gel Check:** Run a 0.7-1% agarose gel to verify the quality of the extraction, if necessary.

✓ ***Important Note***:
The dsRNA concentration will be measured using a high-sensitivity dsDNA Qubit (unofficially confirmed in discussions with Thermo Fisher's Qubit team). After column chromatography and digestion with RNase and DNase, we expect minimal to no RNA and variable amounts of DNA (theoretically dsRNA), typically reading below 2 ng/µl for soil samples.

Buffers Preparation

- **Stock 1M Phosphate Buffer (PB)**
  Prepare the 1M phosphate buffer based on the number of samples and your needs. It can be stored at room temperature for up to 6 months. For this protocol, the working concentration is 240 mM, which will be diluted from the 1M stock.

Compounds	Molecular Weights	Amount for 50 ml (g)	Amount for 200 ml (g)	Concentration (mM)
K_2HPO_4	174.18	6.789	27.156	779.56
KH_2PO_4	136.09	1.5	6	220.44

  - **Preparation**: Dissolve the appropriate salts in water to achieve a portion of the final volume (35 ml for a total of 50 ml or 150 ml for a total of 200 ml).
  - **Check pH**: Measure the pH of the solution; it should be approximately 7.3–7.4.
  - **Adjust pH**: The amounts of each compound are calculated to achieve the target pH without requiring adjustment. However, if needed, adjust the pH to the specified range, and then bring the solution to the final volume of either 50 ml or 200 ml.

- **PVP-Amended CTAB Buffer (PCB)**
  The PCB is prepared by combining two separate solutions: CTAB buffer and PB, in a 1:1 volume ratio. Follow the instructions below to prepare these two components separately, autoclave them, and mix together after sterilization. Note that the final volume of the PCB will be double that of the volume you choose from the table below.

Compounds	Molecular Weights	Amount for 150 ml (g)	Amount for 200 ml (g)	Concentration
NaCl	58.44	16.363	12.272	1.4 M
CTAB	364.45	15	20	10 %
PVP 40,000	40,000	5.1	6.8	3.4 %

- **Preparation:** Preheat the water bath to 65°C. Place a magnetic stir bar in a clean bottle that is double the volume of the solution you are preparing (e.g., use a 400 ml bottle for a 200 ml solution). Add the required compounds, then add water to bring it to the final volume.
- **Dissolve the Compounds:** Place the bottle in the water bath for 15–20 minutes to aid dissolution. Once sufficiently heated, transfer the bottle to a magnetic stirrer and continue stirring until the compounds are fully dissolved. If necessary, repeat the heating and stirring steps to maximize dissolution.
- **Prepare working PB:** In a separate clean bottle, prepare the same volume of phosphate buffer from a 1M stock to achieve a final concentration of 240 mM. Use the following volumes as a reference:

Desired Volume (ml)	Volume from 1M Stock (ml)	Volume of Water (ml)
200	48	152
150	36	114

- **Autoclave:** Keep the magnetic stir bar in the first bottle and autoclave both the CTAB solution and phosphate buffer for 20 minutes at 121°C.
- **Mix Solutions:** While both solutions are still hot, pour the phosphate buffer into the CTAB solution (ensuring there's enough space in the bottle). Stir thoroughly to achieve complete homogenization. Allow the mixture to cool to room temperature before use.

- **PEG-NaCl**
  Use washed, clean, and baked glassware to ensure optimal cleanliness. Select high-quality, molecular biology grade PEG when possible, and always use ultrapure, nuclease-free water for this buffer. If preparing in a highly sensitive area with strict clean procedures, autoclaving may not be necessary. If autoclaving is performed, continue to use ultrapure water, as RNase inactivation through autoclaving is reversible.

Compounds	Molecular Weights	Amount for 200 ml (g)	Concentration
PEG	8000	26	13 %
NaCl	58.44	14	1.2 M

- **Procedure:** Add the necessary compounds and bring the total volume to 150 ml. Shake thoroughly for 15 minutes at 300 rpm, or until fully dissolved, then bring the solution to its final volume. If autoclaved, mix the solution well while still hot. The solution may appear cloudy or show two phases after autoclaving, but it will clear and become transparent after thorough mixing and cooling.

- **Column Chromatography Stock Buffer (STE 10X)**

Compounds	Molecular Weights	Amount for 200 ml (g)	Concentration
Tris-Base	121.14	2.422	100 mM
NaCl	58.44	11.688	1 M
EDTA	292.24	0.584	10 mM

  - **Procedure:** Add the necessary compounds and bring the total volume to 150 ml. Mix thoroughly using a magnetic stirrer until fully dissolved. Adjust the pH to 7.6 using diluted HCl. Once the pH is correct, bring the solution to a final volume of 200 ml, then autoclave for sterilization.

- **Chromatography Washing Buffer**
  Prepare this buffer freshly before starting the lysis step in a 50 ml conical tube, which is sufficient for 8 samples.
  - Water: 37 ml
  - STE 10X: 5 ml
  - Anhydrous Ethanol: 8 ml
  - Mix well before use.

Important Note:
Ensure all buffers are prepared according to the specified concentrations and procedures before proceeding with the extraction protocol.

Abdonaser Poursalavati, Ph.D candidate
Saint-Jean-sur-Richelieu Research and Development Centre, Agriculture and Agri-Food Canada
Department of Biology, Université de Sherbrooke
Abdonaser.poursalavati@canada.ca
Poursalavati.abdonaser@usherbrooke.ca

1. Poursalavati, A., et al., *Soil metatranscriptomics: An improved RNA extraction method toward functional analysis using nanopore direct RNA sequencing.* Phytobiomes Journal, 2023.
2. Poursalavati, A., A. Larafa, and M.L. Fall, *dsRNA-based viromics: A novel tool unveiled hidden soil viral diversity and richness.* bioRxiv, 2023: p. 2023.05.10.540251.
3. Javaran, V.J., et al., *First detection and genomic characterisation of Spinach latent virus in tomato in Canada.* New Disease Reports, 2024. **49**(1): p. e12249.
4. Lussier-Lépine, M., et al., *First Report of Alstroemeria Necrotic Streak Virus Infecting Greenhouse Bell Pepper (Capsicum annuum) in Canada.* Plant Disease, 2023. **107**(8): p. 2562.
5. Drury, S.C., et al., *Exploring the mycovirome: novel and diverse mycoviruses in Botrytis cinerea.* bioRxiv, 2025: p. 2025.03.28.645015.
