# Supplementary material for: SS-VIME: a single-source virome-microbiome extraction protocol toward comprehensive soil community analysis
Source: Microbiol Spectr. 2026 Mar 24;14(5):e03323-25. doi: 10.1128/spectrum.03323-25 (PMC13141886; doi:10.1128/spectrum.03323-25)
Supplement: Figure S1 — Negative Control PCR. Agarose gel electrophoresis of PCR products from the negative control extraction. The input sample was a non-spiked, sterilized mixture of pooled bulk and rhizosphere soil (C1/C2). Lanes verify the absence of amplifiable DNA using 16S rRNA and ITS primers, compared to a no-template control (NTC). L: 100 bp DNA ladder. [file spectrum.03323-25-s0006.docx]

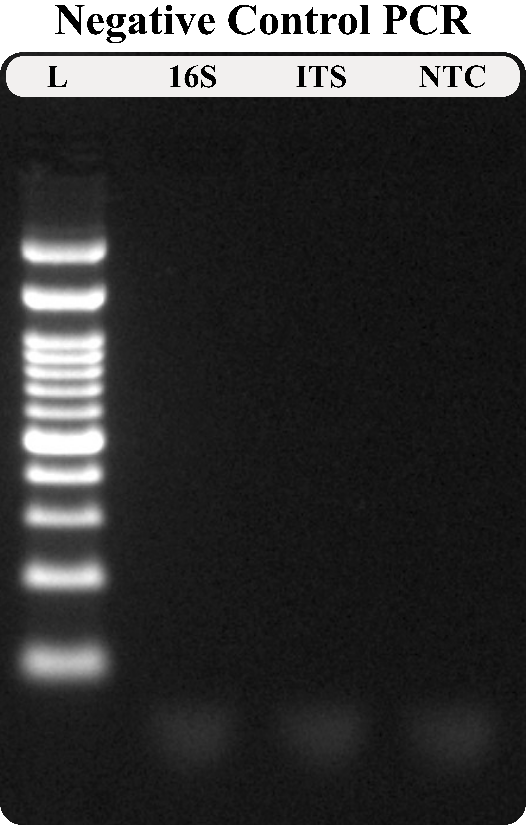


**Supplementary Figure 1. Validation of soil sterilization.** Agarose gel electrophoresis of PCR products from the negative control extraction. The input sample was a non-spiked, sterilized mixture of pooled bulk and rhizosphere soil (C1/C2). Lanes verify the absence of amplifiable DNA using 16S rRNA and ITS primers, compared to a no-template control (NTC). L: 100 bp DNA ladder.
